# Health-Related Quality of Life in Romanian Patients with Dystonia: An Exploratory Study

**DOI:** 10.3390/jcm13123403

**Published:** 2024-06-11

**Authors:** Ovidiu Lucian Băjenaru, Cătălina Raluca Nuță, Lidia Băjenaru, Alexandru Balog, Alexandru Constantinescu, Octavian Andronic, Bogdan Ovidiu Popescu

**Affiliations:** 1 Faculty of Medicine, “Carol Davila” University of Medicine and Pharmacy, 050474 Bucharest, Romania; ovidiu.bajenaru@umfcd.ro (O.L.B.); alexandru.constantinescu@umfcd.ro (A.C.); octavian.andronic@umfcd.ro (O.A.); bogdan.popescu@umfcd.ro (B.O.P.); 2 National Institute of Gerontology and Geriatrics “Ana Aslan”, 011241 Bucharest, Romania; 3 Department: Communications, Applications, and Digital System, National Institute for Research and Development in Informatics—ICI Bucharest, 011455 Bucharest, Romania; lidia.bajenaru@ici.ro; 4 Department of Computer Science, Faculty of Automatic Control and Computers, National University of Science and Technology Politehnica Bucharest, 060042 Bucharest, Romania; 5 Doctoral School of Economic Informatics, Bucharest University of Economics Studies, 010374 Bucharest, Romania; sandubalog@gmail.com; 6 Gastroenterology Department, University Emergency Hospital of Bucharest, 050098 Bucharest, Romania; 7 General Surgery Department, University Emergency Hospital of Bucharest, 050098 Bucharest, Romania; 8 Department of Neurology, Colentina Clinical Hospital, 020125 Bucharest, Romania

**Keywords:** dystonia, health-related quality of life, EQ-5D-5L, level sum score, visual analog scale

## Abstract

**Background/Objectives**: Dystonia is a neurological movement disorder characterized by involuntary muscle contractions that lead to abnormal movements and postures; it has a major impact on patients’ health-related quality of life (HRQoL). The aim of this study was to examine the HRQoL of Romanian patients with dystonia using the EQ-5D-5L instrument. **Methods**: Responses to the EQ-5D-5L and the visual analogue scale (VAS) were collected alongside demographic and clinical characteristics. Health profiles were analyzed via the metrics of the EQ-5D-5L, severity levels, and age groups. Using Shannon’s indexes, we calculated informativity both for patients’ health profile as a whole and each individual dimension. Level sum scores (LSS) of the EQ-5D-5L were calculated and compared with scores from the EQ-5D-5L index and VAS. The HRQoL measures were analyzed through demographic and clinical characteristics. Descriptive statistics, Spearman correlation, and non-parametric tests (Mann–Whitney U or Kruskall–Wallis H) were used. The level of agreement between HRQoL measures was assessed using their intraclass correlation coefficient (ICC) and Bland–Altman plots. **Results**: A sample of 90 patients was used, around 75.6% of whom were female patients, and the mean age at the beginning of the survey was 58.7 years. The proportion of patients reporting “no problems” in all five dimensions was 10%. The highest frequency reported was “no problems” in self-care (66%), followed by “no problems” in mobility (41%). Shannon index and Shannon evenness index values showed higher informativity for pain/discomfort (2.07 and 0.89, respectively) and minimal informativity for self-care (1.59 and 0.68, respectively). The mean EQ-5D-5L index, LSS, and VAS scores were 0.74 (SD = 0.26), 0.70 (SD = 0.24), and 0.61 (SD = 0.21), respectively. The Spearman correlations between HRQoL measures were higher than 0.60. The agreement between the EQ-5D-5L index and LSS values was excellent (ICC = 0.970, 95% CI = 0.934–0.984); the agreement was poor-to-good between the EQ-5D-5L index and VAS scores (ICC = 683, 95% CI = 0.388–0.820), and moderate-to-good between the LSS and VAS scores (ICC = 0.789, 95% CI = 0.593–0.862). **Conclusions**: Our results support the utilization of the EQ-5D-5L instrument in assessing the HRQoL of dystonia patients, and empirical results suggest that the EQ-5D-5L index and LSS measure may be used interchangeably. The findings from this study highlight that HRQoL is complex in patients with dystonia, particularly across different age groups.

## 1. Introduction

Dystonia is a complex neurological disorder characterized by involuntary muscle contractions that can lead to abnormal movements and postures [1,2]. It can manifest as an isolated condition or alongside other neurological disorders such as stroke [3], Parkinson’s disease (PD) [4], and multiple sclerosis (MS) [5]. Dystonia can occur post-stroke, as an early sign of PD, or as a side effect of PD treatments like levodopa. In MS patients, dystonia complicates the management of symptoms and significantly impacts the quality of life. Treatment options include pharmacotherapy, botulinum toxin injections, and rehabilitation services. For patients suffering with movement disorders, comprehensive neurological evaluations are essential for improving our understanding of the links between such disorders. Advances in research, including the use of artificial intelligence and smart devices, aim to improve diagnostic processes, treatment efficacy, and patient outcomes [6,7]. Accurate phenotyping, classification, and novel diagnostic algorithms are crucial for personalized and effective treatments that will ultimately enhance the quality of life of those affected [8,9].

Dystonia is a multifaceted movement disorder characterized by a wide range of symptoms and significant effects on patients’ quality of life. Understanding its various causes—whether idiopathic, genetic, resulting from injury, or drug-induced—is vital to the creation of effective treatment and management approaches. Providing comprehensive care that addresses both motor and non-motor symptoms is essential as we seek to improve the well-being of those affected by dystonia [1,10].

Studying the health-related quality of life (hereafter, HRQoL) of patients with dystonia is essential for the comprehensive understanding of the disorder’s effects, guiding patient-centered clinical management, informing healthcare policy, and contributing to a more holistic approach to neurological disease research that prioritizes patient well-being and quality of life [8]. This broader perspective is crucial for developing effective interventions and support systems that address the needs of individuals with dystonia and other neurological conditions [11].

Investigating HRQoL in patients with dystonia is essential due to the complex nature of this neurological disorder and its significant impact on daily living [6]. The involuntary muscle contractions specific to this disease significantly affect the physical, psychological, and social well-being of patients [12]. Some critical elements of dystonia influence HRQoL and thus indicate the importance of studying HRQoL in the broader context of neurological diseases; these elements may take the form of motor symptoms (muscle spasms, tremors, and involuntary movements); non-motor symptoms (psychological conditions such as anxiety, depression); pain and discomfort; and difficulties in social interaction and employment [11].

In Europe, the study of dystonia and its impact on the quality of life has been robust, being supported by a network of research institutions, hospitals, and patient organizations. European researchers have contributed significantly to our collective understanding of dystonia’s genetic factors, with several studies focusing on the prevalence, diagnosis, and management of dystonia in different European populations. The European Dystonia Federation, for example, plays a pivotal role in raising awareness, supporting research, and advocating for patient care across the continent [13,14].

In Romania, the medical community is increasingly recognizing the significance of neurological disorders including dystonia [15,16]. An analysis of the quality of healthcare in Romania compared to other countries will help to contextualize the specific challenges of managing dystonia within the Romanian healthcare system [17].

The EQ-5D, developed by the EuroQol Group [18], is used to assess the quality of life in patients with neurological disorders. The five-level version (EQ-5D-5L) introduced in 2009 evaluates five dimensions of health-related quality of life: mobility, self-care, usual activities, pain/discomfort, and anxiety/depression [19]. It also includes a visual analog scale (EQ VAS) that provides a quantitative measure of health outcomes based on patients’ perceptions [20].

Upon searching PubMED, ScienceDirect, and Web of Science, we identified only a few studies published in the last five years that used EQ-5D-5L in patients with dystonia. One multicenter observational study conclusively demonstrated that benign essential blepharospasm (BEB), a form of dystonia characterized by involuntary muscle contractions around the eyes, significantly impairs the quality of life (QoL) of affected individuals. This impact was evident in several areas such as mobility, usual activities, pain/discomfort, and anxiety/depression, all measured by the EQ-5D-5L instrument. The utility of using the EQ-5D-5L as an assessment of the QoL lies in its ability to effectively capture these effects [21]. The aforementioned study’s findings underscore the efficacy of botulinum toxin type A (BTX-A) in alleviating the debilitating effects of BEB, supporting its inclusion in health coverage policies to ensure broader access and improved patient outcomes in Thailand.

A large cross-sectional study from China explored the impact of cervical dystonia (CD) on patients’ quality of life using the EQ-5D-5L instrument. The study involved 333 patients and found that the most frequently affected dimensions of health were anxiety/depression, pain/discomfort, and usual activities. It highlighted the significant role of non-motor symptoms such as depression, pain, and sleep quality in determining QoL in patients with CD. The EQ-5D-5L as an evaluator of the QoL was shown to be effective in describing these symptoms, underscoring its utility in assessing the health-related quality of life in patients with cervical dystonia [22].

Another study validated the EQ-5D-5L’s capacity to assess the quality of life in acute stroke patients through a cross-sectional analysis of 408 participants [23]. Compared to the EQ-5D-3L, the EQ-5D-5L showed improved discriminatory power, especially in the dimensions of pain/discomfort and anxiety/depression. It demonstrated feasibility, construct validity, and strong convergence with established stroke measures like the modified Rankin Scale and the Barthel Index of patient care. The effectiveness and utility of the EQ-5D as a comprehensive tool for HRQoL assessment was also demonstrated in the study [24], providing valuable insights in health assessments and informing policy decisions.

Maione and colleagues [10] explored how non-motor symptoms (NMSs) significantly affect the quality of life of patients with cervical dystonia (CD). The authors reviewed studies from major databases and found that NMSs are critical predictors of a decline in the QoL, often more so than motor symptoms. They thus indicate the need for a comprehensive approach to managing CD that includes the NMS approach to improve patient well-being; the authors also recommended that further research into these symptoms be carried out to improve clinical management and treatment outcomes.

The efficacy of botulinum toxin treatment in improving the quality of life of dystonia patients has been proven by measuring changes in EQ-5D-5L scores, with a particular focus on the dimensions of physical mobility and self-care [25]. The effects of botulinum toxin (BoNT) injections on the health-related quality of life in patients on the spectrum of complex spasticity were analyzed using the EQ-VAS and EQ-5D-5L. The results showed that BoNT improved the EQ visual analog scale (EQ VAS) scores. In addition, the health status and pain maxima improved.

The literature indicates a significant association between dystonia and the presence of anxiety and depression. The chronic nature of the disorder, along with the unpredictability of symptoms and societal stigma, can lead to psychological distress, further impacting patients’ quality of life. Non-motor symptoms, namely psychological distress (anxiety and depression) and deficiencies in cognitive and social functioning, are among the distinct characteristics of cervical dystonia [26]. Depression in patients with focal-onset idiopathic dystonia has been shown to contribute to the worsening of disease symptoms [27,28].

Numerous studies have emphasized the significant impact of idiopathic dystonia on the quality of life, the efficacy of treatments such as botulinum toxin and deep brain stimulation in enhancing it, and the critical importance of routinely assessing and monitoring quality of life in patients with dystonia for informing management and treatment decisions [29].

The outcomes of these studies have shown the utility of QoL assessment in clinical practice; moreover, they suggest measures to be implemented by professionals that may induce an increase in the QoL. Although dystonia research has advanced significantly in recent years, several gaps remain in our current understanding and management of the condition. Such gaps mainly exist in our understanding of the full impact of dystonia beyond its motor symptoms and may be remedied by integrating patient perspectives into care and encouraging international collaboration for optimal research and treatment approaches. Comprehensive understanding of non-motor symptoms and their impact on health and the associated HR-QoL is a crucial consideration.

This paper substantially expands upon a previous work [30] that presented a shorter analysis of the impact of dystonia on the quality of life of patients in Romania.

## 2. Materials and Methods

### 2.1. Study Design

This study was conducted between May and September 2021, and included 90 patients with idiopathic dystonia and hemifacial spasm from the Neurology Clinic of the Colentina Clinical Hospital, Bucharest, Romania. Patients with idiopathic dystonia were included in the study to ensure a homogeneous population, allowing for a more accurate assessment of both the quality of life and consequences of treatment. Idiopathic dystonia, which lacks an identifiable cause, presents a more consistent clinical pattern when compared with functional dystonia. This allows for a more accurate assessment of the quality of life and consequences of treatment. The reason for including both patients with idiopathic dystonia and those with hemifacial spasm was to understand how patients with movement disorders at this tertiary center in Bucharest perceive their quality of life. While both conditions involve involuntary muscle contractions, they are distinct disorders with different underlying mechanisms and clinical presentations. However, they both share botulinum toxin as a common treatment. This study was cross-sectional, and patient anonymity and data protection were ensured. The Ethics Committee of the Clinical Hospital Colentina, Bucharest, approved the study, and the selected patients were enrolled only after signing informed consent.

Until now, in Romania, there has been no official registry of patients diagnosed with dystonia. Thus, there are no population-level data on the extent of the dystonic phenomenon for public authorities to use in formulating health policies (both for pandemic situations and everyday life). We also lack precise data regarding which medical centers (hospitals or specialty outpatient clinics) may be able to provide botulinum toxin to patients with dystonia and associated conditions such as hemifacial spasm. However, the Neurology Clinic of the Colentina Clinic Hospital, Bucharest (where the study took place) is a specialized tertiary center that offers botulinum toxin injection services to patients with movement disorders.

Neurologists followed a systematic approach to diagnosing idiopathic dystonia, which included clinical assessment, diagnostic criteria as established by the Movement Disorder Society (MDS), and various tests [2]. The following inclusion criteria were established for this study: age over 18 years; spoken Romanian language; a definite diagnosis of dystonia or hemifacial spasm, regardless of the type of dystonia or duration of treatment before the start of the study; written informed consent from the patient or their legal representative; and absence of any disability that could affect self-administration of study questionnaires during hospital visits [31].

The research team administered the study questionnaire via face-to-face interviews with 90 patients diagnosed with dystonia. Positive diagnoses were established by the neurologist based on clinical and paraclinical elements, and management was differentiated based on individual characteristics and in accordance with the dystonia criteria set by the MDS [2]. Approval for the use of the EQ-5D-5L questionnaire was granted by the EuroQoL Research Foundation.

The questionnaire consisted of the following sections: demographic questions, dystonia-related characteristics, and the EQ-5D-5L instrument (comprising a descriptive system and visual analogue scale). Demographic questions included sex, age at the beginning of the survey, age of onset, comorbidities, type of disorder, time to diagnosis, and disease duration. Dystonia-related characteristics encompassed clinical characteristics (such as body distribution and temporal patterns) and etiology (nervous system pathology or idiopathic quality), according to methodological recommendations [1,2].

Participants were asked to rate their health on a five-point scale, with 1 indicating no problems and 5 indicating extreme problems. Patients indicated their health status by choosing the most appropriate statement from each of the five dimensions. These decisions resulted in a one-digit number. The numbers for the five dimensions could then be combined into a five-digit number that described the patient’s health status [19]. Participants were also asked to self-rate their current health status using a visual analog scale (VAS), where 0 represents the “worst possible health status” and 100 represents the “best possible health status” [18].

Data collected from the descriptive system (health states) were converted to an EQ-5D-5L index using the Romanian value set [32]. This index reflects societal preferences for various states of health and ranges from −0.323 to 1.000, where negative values indicate poor health conditions (states worse than death) and 1.000 represents perfect health [32]. To obtain an overall score for each health state and approximate overall health status, dimension-level responses were summed to produce a level sum score (LSS). Ranging from 5 (11111, indicating no problems in any dimension) to 25 (55555, indicating extreme problems in all dimensions), the LSS reflects poorer health with higher scores [33,34].

**Figure 1:**
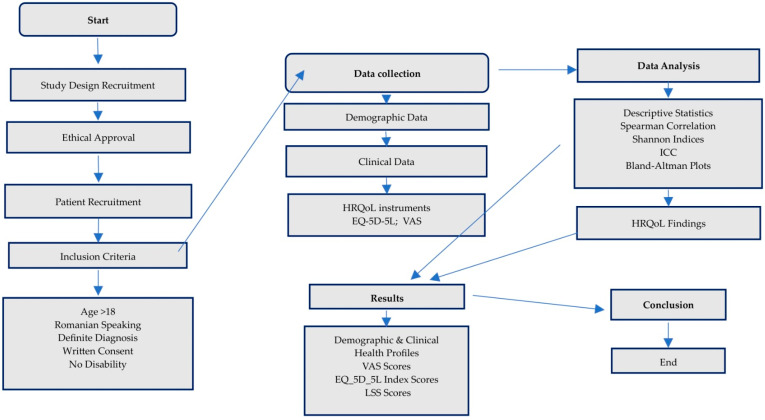
Study methodology and key processes flowchart.

The flow diagram based on the study in this paper can be seen in Figure 1. It represents the key stages and processes involved in the study, starting with the introduction and the importance of the study, followed by obtaining ethical approval. Patient selection is based on inclusion criteria such as age, language, diagnosis, consent, and ability to complete questionnaires. Data collection includes demographic data, clinical data, HRQoL measures, and the EQ-5D-5L and VAS questionnaires. These data are subjected to variable analysis and data processing, branching into descriptive statistics. The study assesses the impact of dystonia on patients and analyzes HRQoL findings, leading to the presentation of results and conclusions and concluding the study process.

### 2.2. Data Analysis

Descriptive statistics were used to characterize patients’ demographic and clinical characteristics, their health profile, and continuous HRQoL variables (VAS, EQ-5D-5L index and LSS). Categorical variables were reported as frequencies and percentages. Fisher’s exact test was used to analyze categorical variables.

Continuous variables were evaluated if they were normally distributed upon Shapiro–Wilk testing and were reported as means (M), standard deviations (SD), median (Mdn), and interquartile range (IQR). Preliminary checks regarding the selection of the types of tests for the continuous variables in correlation with the size of the groups related to the categorical variables indicated that non-parametric tests were appropriate for use in this study. Thereafter, continuous variables were analyzed with the Mann–Whitney U and Kruskal–Wallis H tests for two groups and three groups, respectively. Correlations between variables were calculated using Spearman’s rank correlation coefficient (*rho*).

Health profiles were reported as frequencies and percentages and were analyzed for each dimension by age group for all patients and separately for males and females. Results were categorized by predefined age-groups (≤54; 55–64; and ≥65 years).

We calculated the informativity of both the descriptive system (health profile) as a whole and each dimension of the EQ-5D-5L using Shannon’s index (absolute informativity, H′) and Shannon’s evenness index (relative informativity, J′), as described in [18,24]. The Shannon index (H′) ranged from 0 to 2.32 for EQ-5D-5L, and the Shannon evenness index (J′) ranged from 0 to 1. A higher H′ value indicates that responses to the dimension are more evenly spread across the different categories (i.e., severity levels) of the dimension and consequently suggest greater informativity. The Shannon evenness index (J′) reflects the extent to which information is uniformly distributed across levels included in the descriptive system.

To compare the results with the EQ-5D-5L index, we presented all VAS values transformed to a scale from 0 to 1 (VAS divided by 100). Additionally, LSS values were transformed from 5–25 to 0–1 so that higher values correspond with better health [33]. Pairwise agreement between the HRQOL variables was examined using the intraclass correlation coefficient (ICC) [35,36] and Bland–Altman plots [37]. The ICC values and 95% confidence interval were calculated using a two-way mixed-effects model with absolute agreement. The 95% confidence interval of the ICC estimate was interpreted according to the following limits: “poor” (<0.50), “moderate” (between 0.5 and 0.75), “good” (between 0.75 and 0.9), and “excellent” (>0.90) [35]. Bland–Altman plots were utilized to illustrate the agreement between pairs of HRQoL measures. These plots display the relationship between the means of scores on the X-axis and the differences between scores on the Y-axis. Three lines on the plot indicate the mean of differences and the limits of agreement (calculated as the mean difference ± 2 × SD). Good agreement is indicated when the mean difference is close to zero and falls within the 95% limit of agreement [38]. Differences that were within the 95% limit of agreement showed that the two HRQoL measures could be used interchangeably.

Statistical analysis was conducted using IBM SPSS Statistics software, version 24.0, with the significance level set at *p* ≤ 0.05 (two-tailed).

## 3. Results

### 3.1. Demographic and Clinical Characteristics

A total of 90 patients completed the questionnaire. The descriptive statistics of demographic variables are provided in Table 1, showing that out of a total of 90 participants, 68 (approximately 76%) were females, and the mean age of the included patients was 58.7 years (SD = 12.6). The female patients were older than the male patients; 70% were older than 54 years in comparison to 55% of males. The mean age of onset was 48.8 years (SD = 12.1), and the mean disease duration was 9.9 years (SD = 7.7). Most patients (80%) reported having dystonia and 74% indicated having comorbidities, the most common of which were cardiovascular diseases (54%) and psychological disorders (7%).

Spearman’s correlation (*rho*) was computed to assess the relationships between age at the beginning of the survey, age at onset and disease duration. Results indicated that there was a significant, strong, and positive correlation between age at the beginning of the survey and age of onset (*rho* = 0.76, *p* < 0.001) and a significant, moderate, and positive correlation with disease duration (*rho* = 0.39, *p* < 0.001). The correlation between age of onset and disease duration was negative and not statistically significant (*rho* = −0.16, *p* = 0.122). The results of the Fisher’s exact test do not indicate a significant association between comorbidities and the type of disorder (*p* = 0.395), between the sex groups and comorbidities (*p* = 0.780), or between sex groups and the type of disorder (*p* = 0.999).

The Mann–Whitney U tests showed that there are no statistically significant differences (*p* > 0.05) between the sex groups regarding age at the beginning of the survey (*p* = 0.372) and disease duration (*p* = 0.832), whereas there are statistically significant differences between the sex groups regarding age of onset (*p* = 0.045). The mean age of onset of female patients was higher than the mean of male patients.

For the whole sample, non-parametric tests showed statistically significant differences (*p* < 0.05) between age at the beginning of the survey and comorbidities (*p* < 0.001) and type of disorder (*p* = 0.012). Additionally, we observed significant differences between age at onset and comorbidities (*p* = 0.001) and type of disorder (*p* = 0.020), respectively. Older patients and those with a higher age of onset of the disease had more comorbidities; dystonia was the most common type of movement disorder. On the other hand, we found no statistically significant differences between the duration of disease and comorbidities (*p* = 0.255) and type of disorder (*p* = 0.796).

When reporting the characteristics of dystonia, more than half of patients described the disease starting in early adulthood (21–40 years). Focal dystonia was the most frequent type of dystonia (87% of patients) and progressively worsened (77%) with symptoms persisting throughout the day without fluctuation (78%). More detailed information on the characteristics of dystonia is provided elsewhere [29,30,31].

### 3.2. Health Profiles

Across the whole sample, the number of different states of health reported by patients was 65 (2.1%) out of a possible 3125, and 12 health states reported by at least 2% of patients represented 40.9% of respondents (Table 2). The other 53 health states were reported only once, including the worst health state (i.e., 55555). The proportion of patients reporting no problems in all five dimensions (i.e., health state 11111) was 10% and was higher in male patients (18.2%) than in female patients (7.4%). The next four most frequently reported health states were 11121 (5.6%), 11122, 21233, and 33333 (with each of these representing 3.3%).

The frequencies and proportions of the EQ-5D-5L dimensions and their level within the total sample are presented in Table 3. Among the 90 patients with dystonia, the highest frequency reported was “no problems” in self-care (66%), followed by the same in mobility (41%), anxiety/depression (36%), usual activities (33%), and pain/discomfort (18%). Pain/discomfort was the dimension most frequently reported by patients as “extreme”, alongside low health status (8%). The other dimensions were reported by patients as “extreme/unable to” in proportions of approximately 6%.

Table 4 shows the frequency of reported problems for each dimension across different age groups for the entire sample, while Table 5 displays the results separately for male and female patients.

For the whole sample, problems with pain/discomfort and the activities of daily living were the most frequent, with 90.3% of patients reporting problems (slight to extreme) in the ≤54 year age group, while problems with self-care were the least frequent, with 24.3% of patients reporting problems in the 65+ year age group. The percentage of reported health problems (any problems) decreased with age in each dimension, except for the pain/discomfort dimension. In this dimension, the percentage of health problems was at the highest level in the ≤54 year age group (90.3%), after which it decreased in the 55–64 year age group (77.3%) and increased slightly in the 65+ year age group (78.4%).

A similar pattern was observed for female patients with the exception of the anxiety/depression dimension, which increased slightly from 71.4% in the ≤54 years age group to 72.2% in the 55–64 year age group, after which it decreased substantially to 55.2% in the 65+ year age group. In male patients, the pattern appeared different. In four dimensions, the reported frequency of problems decreased from the ≤54 year age group to the 55–64 year age group, while such frequency increased from the 55–64 year age group to the 65+ year age group. The exception was the anxiety/depression dimension, for which the frequencies were identical for the older age groups.

Both the Shannon index and the Shannon evenness index showed greater informativity for the pain/discomfort dimension (2.17 and 0.89, respectively) and the least informativity for the self-care dimension (1.59 and 0.68, respectively) (Table 6). Taking sample size (N = 90) into account, we found many unique states of health (65). Participants’ observations were less concentrated in a small number of states, indicating that the patients were heterogeneous in terms of health profile.

### 3.3. EQ VAS

**Figure 2:**
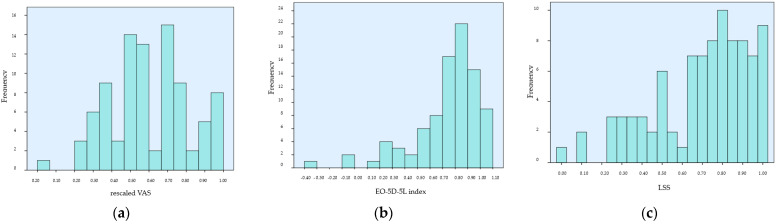
Distributions: (**a**) rescaled VAS; (**b**) EQ-5D-5L index; (**c**) level sum score.

Figure 2a shows the frequency distribution of VAS scores. The rescaled VAS scores ranged from 0 to 1.00, with the three most frequently reported scores being 0.70 (16.7%), 0.50 (15.6%) and 0.60 (11.1%). The proportion of VAS scores reaching 0.60 was 54.4% (49 patients); 31.1% (28 patients) of participants reported scores between 0.61 and 0.85, and 14.5% (13 patients) reported scores between 0.86 and 1.00 (including 2.2% (two patients) who reported the maximum score of 1.00). Fewer than half of the patients (approximately 46%) had a VAS score > 0.60.

VAS descriptive statistics for the total sample are presented in Table 7. The mean score of VAS for the total sample was 0.61 (SD = 0.21). The results of the Mann–Whitney U test indicated that there was no significant difference between the VAS scores of male patients (mean rank of 43.93) and female patients (mean rank of 46.01), *Z* = −0.326, *p* = 0.745. There was also no statistically significant difference in VAS scores between age groups at the start of the survey (χ^2^(2) = 0.041, *p* = 0.980); we observed a mean rank VAS score of 44.85 for the age group ≤ 54 years, 46.32 for the age group 55–64 years, and 45.55 for the age group 65+ years. Concerning age at onset and the disease duration, Kruskal–Wallis H tests indicated that there were no statistically significant differences in VAS scores between groups (χ^2^(2) = 4.420, *p* = 0.110 and χ^2^(2) = 5.557, *p* = 0.062, respectively).

In addition, the results of the Mann–Whitney U test indicated that there was a significant difference between the VAS scores of patients with dystonia (mean rank of 42.81) and patients with hemispasm (mean rank of 56.25), *Z* = −1.964, *p* = 0.049. Concerning comorbidities, the Kruskal–Wallis H test indicated significant difference (χ^2^(2) = 7.09, *p* = 0.029) between the mean ranks of at least one pair of groups. Dunn’s pair-wise tests were conducted for the three pairs of groups. There was strong evidence (*p* = 0.029, adjusted using Bonferroni correction) of a difference in VAS scores between patients without comorbidities (mean rank of 54.85) and those with two or more comorbidities (mean rank of 36.64). No significant differences were found between the other pairs.

### 3.4. EQ-5D-5L Index

The distribution of the EQ-5D-5L scores skewed left, as illustrated in Figure 2b, and values ranged from −0.323 to 1.000. The proportion of EQ-5D-5L values inclusive of up to 0.60 was 21.1% (19 patients); 40.0% (36 patients) of participants recorded scores between 0.61 and 0.85, and 38.9% (35 patients) recorded scores between 0.86 and 1.00, including 10.0% (nine patients) who recorded the maximum score of 1.00. More than two thirds of patients (approximately 79%) had an EQ-5D-5L index > 0.60.

As shown in Table 7, the mean EQ-5D-5L index score across the entire sample of patients was 0.74 (SD = 0.26). The results of the Mann–Whitney U test indicated that there was no significant difference between the EQ-5D-5L index scores of male patients (with a mean rank of 44.68) and female patients (with a mean rank of 45.75), *Z* = −0.169, *p* = 0.869. Additionally, the Kruskal–Wallis H test indicated that there was no statistically significant difference in EQ-5D-5L index scores between groups with various comorbidities (χ^2^(2) = 2.750, *p* = 0.253); we observed a mean rank of 49.78 for patients without comorbidities, of 48.38 for patients with one comorbidity, and of 39.55 for patients with two or more comorbidities.

The results indicated significant differences between the EQ-5D-5L index values of patients with dystonia (with a mean rank of 41.49) and patients with hemispasm (a mean rank of 61.53): *Z* = −2.912, *p* = 0.004. Duration of disease also showed significant differences: χ^2^(2) = 7.42, *p* = 0.024 (*p* = 0.020, adjusted using the Bonferroni correction).

Regarding age at the start of the survey, the Kruskal–Wallis H test provided evidence of a difference (χ^2^(2) = 7.40, *p* = 0.025) between the mean ranks of at least one pair of groups. Dunn’s pair-wise tests were carried out for the three pairs of groups. There was strong evidence (*p* = 0.020, adjusted using the Bonferroni correction) of a difference between the ≤54 year age group (with a mean rank of 36.31) and the 65+ year age group (a mean rank of 53.58). There was no evidence of a difference between the other pairs.

With respect to the age at onset, the Kruskal–Wallis test revealed a statistically significant difference in EQ-5D-5L index score among the different groups, χ^2^(2) = 7.82, *p* = 0.020. Strong evidence (*p* = 0.018, adjusted using the Bonferroni correction) indicated a difference between the group aged 41–54 years at onset (mean rank of 38.54) and the group aged 55+ years at onset (mean rank of 56.09). No significant differences were found between the other pairs.

### 3.5. Transformed Level Sum Score (LSS)

The LSS was determined by summing the level scores for the five dimensions and then performing a linear transformation on this total to a 0–1 scale. In this transformation, a score of 11111 corresponds to 1.0, and a score of 55555 corresponds to 0, with a higher score indicating better health [33]. The distribution of the LSS values was left-skewed, as shown in Figure 2c. The proportion of LSS values inclusive of up to 0.60 was 28.9% (26 patients); 55.6% (50 patients) recorded scores between 0.61 and 0.85, and 26.7% (24 patients) recorded scores between 0.86 and 1.00. More than two thirds of patients (approximately 82%) had an LSS score > 0.60.

As shown in Table 7, the mean LSS for the entire sample of patients was 0.70 (SD = 0.24). The pattern of differences between groups was similar to that of the EQ-5D-5L index. There were no statistically significant differences between the male patients and female patients (*Z* = −0.033, *p* = 0.974) or between groups with various comorbidities (χ^2^(2) = 7.09, *p* = 0.029). In addition, the results indicated that there were significant differences between the LSS scores in the following groups: type of disorder (*Z* = −2.990, *p* = 0.003), disease duration (χ^2^(2) = 7.09, *p* = 0.029), age at the start of the survey (χ^2^(2) = 7.56, *p* = 0.023), and age at onset (χ^2^(2) = 7.53, *p* = 0.023).

### 3.6. Associations and Agreement between HRQOL Measures

The Spearman correlations (*rho*) between the three HRQoL measures were positive and statistically significant (*p* < 0.01), indicating a good degree of association (Table 8). There was a very strong correlation between the EQ-5D-5L index and LSS scores (0.99). Additionally, we found strong correlations between the EQ-5D-5L and VAS and between the LSS and VAS, 0.62 and 0.64, respectively.

The level of agreement between the pairs of HRQoL measures varied, as indicated in Table 8. For instance, the agreement between the EQ-5D-5L index and LSS values was excellent, showing an ICC of 0.970 (95% CI = 0.934–0.984); that said, we observed only poor to good agreement between the EQ-5D-5L index and VAS scores, with an ICC of 0.683 (95% CI = 0.388–0.820). Additionally, there was moderate to good agreement between the LSS values and VAS scores.

**Figure 3:**
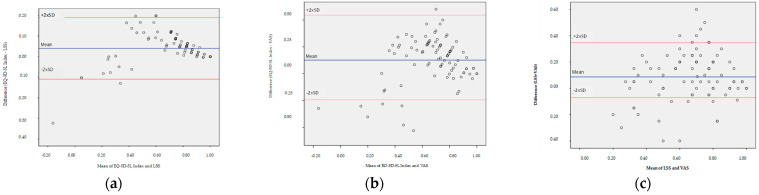
Bland–Altman plots of the difference in scores between (**a**) EQ-5D-5L and LSS, (**b**) EQ-5D-5L and VAS, and (**c**) LSS and VAS. The horizontal blue line represents the mean of the differences in scores, while the 95% confidence interval is represented by red lines. Table 8 summarizes the mean difference and the upper and lower 95% limits of agreement as well as their 95% confidence intervals.

Bland–Altman results are reported in Table 8 and illustrated in Figure 3. Analysis showed a mean difference of 0.04 units (95% limits of agreement: −0.11 to 0.19) between EQ-5D-5L index and LSS score; a difference of 0.12 units (95% limits of agreement: −0.30 to 0.55) between EQ-5D-5L index and VAS score; and a difference of 0.09 units (95% limits of agreement: −0.28 to 0.46) between LSS and VAS scores. The limits of agreement were broad; the narrowest limits of agreement (0.30) could be observed for paired EQ-5D-5L and LSS scores. Narrower limits of agreement indicated more precision. The Bland–Altman plots (Figure 3) illustrate that 95.6%, 91.1%, and 79.9% of observed differences were within the limits of agreement. EQ-5L-5L index scores were higher in 82.2%, 78.9%, and 77.8% of cases, respectively.

The EQ-5D-5L index and LSS showed a high level of agreement for the whole sample. However, the results indicated relatively large differences at the individual level (Figure 3a), particularly for lesser states of overall health (where EQ-5D-5L index values are lower than 0.70). For the other pairwise comparisons, Bland–Altman plots (Figure 3b,c) indicated larger individual differences with broader limits of agreement (0.84 and 0.73, respectively).

## 4. Discussion

This study, using the EQ-5D-5L instrument at a neurology clinic, addresses the gap in our understanding and reporting of health-related quality of life in patients with dystonia, a topic not previously reported in Romania or other countries.

The findings showed that dystonia patients reported problems across various dimensions of the EQ-5D-5L, ranging from “slight” to “extreme”. The highest frequency of issues was reported in the pain/discomfort dimension (82.2%), while the lowest was in the self-care dimension (34.4%). Overall, more than half of the patients reported problems in all dimensions except for self-care, indicating poor perceptions of their own health. The ordered sequence of the most reported health problems (pain/discomfort, usual activities, anxiety/depression, mobility, and self-care) showed similarity to the findings of a previous study [22] that reported the highest frequencies in the dimensions of anxiety/depression (73.6%) and pain/discomfort (68.2%).

Pain is an important feature of dystonia, resulting from continuous muscle contractions and excessive strain on musculoskeletal structures and leading to fatigue, tension, and the deterioration of muscle function. Compared to other dimensions of the quality of life, such as mobility, self-care, daily activities, and psychosocial problems (anxiety and depression), pain and discomfort have a more pronounced impact. Studies using instruments other than the EQ-5D-5L to measure quality of life have shown that patients with dystonia describe pain as their main concern, placing greater importance on it than other aspects such as mobility or anxiety/depression [39,40].

Although mobility and self-care are often impaired in dystonia, its impact on the quality of life can be mitigated through strategies and treatments. In contrast, pain is more difficult to control and can have a cascading effect on individuals’ emotional state and ability to maintain daily activities, thereby intensifying feelings of anxiety and depression.

In the context of dystonia, research has generally shown that while pain, mobility, and social participation are significantly affected, patients may experience less difficulty in self-care. This is due to the nature of dystonia, which, depending on its type and severity, may not always severely limit an individual’s ability to perform personal care tasks [38].

Our results indicated that approximately 90% of patients who reported problems (any problems) with pain/discomfort and usual activities were in the age group ≤ 54 years. Problems with self-care were the least frequent, with 24.3% of patients reporting problems in the 65+ year age group. Surprisingly, except for the dimension of pain/discomfort, the percentage of reported health problems (any problems) decreased with age in each dimension. However, age-related patterns differed between male patients and female patients. Problems were reported more frequently by female patients than male patients across all dimensions, with the most significant difference observed in the aspect of self-care (35.3% vs. 31.8%) and the performance of usual activities (67.6% vs. 63.6%). These findings are in contrast with those of previous studies that have reported an increase in the percentage of problems (of any kind) reported via the EQ-5D-5L [41].

Research and clinical observations suggest several factors that may contribute to the improvement or variation in dystonia symptoms over time, including age at onset, comorbidities, and motor and non-motor symptoms. Also among these factors are genetic factors and comorbidities (such as Wilson’s disease [42,43], which in our study’s case was impossible to investigate). Each of these factors underscores the complexity of dystonia as a condition and the need for individualized approaches to treatment. The variability in the progression of symptoms and patients’ response to treatment highlights the importance of comprehensive diagnostic evaluations that include genetic testing and careful consideration of the patient’s history and symptoms upon presentation.

Closely related to these findings, the Shannon index and Shannon evenness index indicated greater informativity in the pain/discomfort dimension and less informativity in the dimension of self-care. Considering the sample size (N = 90), we found a large number of unique health states (65), indicating that the patients were heterogeneous in terms of their health profiles. The dispersion of patients’ profile data across numerous profiles suggests that individualized treatment approaches may be necessary. Our observations of different EQ-5D-5L health profiles emphasize the complexity of HRQoL in dystonia patients, particularly across different age groups.

Our results show that dystonia has an important impact on the HRQoL in Romanian patients, with a mean EQ-5D-5L index of 0.74, a mean VAS score of 0.61, and a mean LSS of 0.70.

In this study, the mean EQ-5D-5L index (0.74) was lower than the overall value for the general Romanian population (0.82), as reported in [32]; in addition, the mean was higher for females (0.75) than for males (0.70). This suggests that patients with dystonia have lower HRQoL than the average Romanian. When comparing our results with those of previous studies, the mean EQ-5D-5L index was approximately similar to that observed in a Mexican study of patients with Parkinson’s disease (0.71) [44] and in a Japanese study of patients with systemic sclerosis (0.74) [45]. However, the mean was considerably higher than the value of 0.58 reported in people with multiple sclerosis from New Zealand [46] and Australia [47]. The mean was lower than that in a study from the USA focusing on patients with oromandibular dystonia, (0.81) [48] and in a study from China focusing on patients with cervical dystonia (0.80) [22]. These differences in scores might be due to differences in preference-based value sets, socio-economic context, older age at onset, and longer durations of disease in this study. Further research comparing patients with dystonia and patients with other neurological diseases is needed.

The mean VAS (unscaled) value in this study (61.1) was considerably lower than found in other studies (73.8 [44], 69.2 [45], 70.0 [49], 70.2 [22], 69.4 [46], and 68.7 [47]). In our study, the EQ VAS score was higher for females (61.7) than for males (59.3). It should be noted that the maximum score for the VAS (1.00, perfect health) was reported by only two patients out of a total of nine patients who recorded the maximum value (1.000) for the EQ-5D-5L index. This result aligns with observations made in [50], where some respondents who reported no problems in any of the EQ-5D-5L dimensions gave a VAS health rating of less than 1.00. These differences in scores may be due to the type of disorder and the high frequency of comorbidities in dystonia patients in our study.

We observed that the VAS measure resulted in lower mean scores than the EQ-5D-5L index scores, which confirms the findings of a relatively recent review in patients with neurological disease [51]. As noted by authors, “the two measures should not be directly compared as they reflect different preferences: while the index value reflects how good or bad the health state is according to the preferences of the general population of a country/region, VAS reflects patient’s subjective values of a health state” [51].

Regarding the LSS measure, the mean score was lower than the EQ-5D-5L index score, but higher than the VAS score. Associations between LSS values and demographic and clinical characteristics followed a similar pattern to that of EQ-5D-5L index values, including increases and/or decreases in relation to age groups. The EQ-5D-5L index and LSS measures had the ability to detect differences between subgroups such as age at the beginning of the survey, age of onset, type of disorder, and disease duration. Nevertheless, only the VAS presented significant differences between patients without comorbidities and patients with two or more comorbidities.

A recent study [34] demonstrated the feasibility of collating the dimensions of the EQ-5D-5L into a level summary score using nonparametric item response theory. Many other previous studies have concurrently analyzed the EQ-5D-5L index and LSS [52,53,54]. As noted by [34], the LSS has both advantages (e.g., simplicity, no algorithm required to estimate the LSS) and limitations (e.g., “two patients may have the same LSS score, but one may have extreme problems in a single dimension, whereas the other may have slight problems in several dimensions”).

We observed that the mean EQ-5D-5L index and LSS values increased from 0.66 and 0.62 (age group ≤ 54 years) to 0.80 and 0.77 (age group 65+ years), respectively. The mean VAS was approximately similar in each age group (0.60, 0.62, and 0.61, respectively). This pattern was not so different from results gleaned from samples of the US adult general population [55,56] as well as a sample of patients with acute stroke in Poland [23]; in such samples, a lower EQ-5D-5L score was observed in patients up to 60 years of age when compared with patients aged 61–70 years. This result differs from the previous studies. In this study, such a pattern may be explained by the lower reported degrees of pain/discomfort and self care with increasing age and sets of values. Furthermore, it is possible that this finding results from health problems in patients with dystonia that are not covered by the five dimensions of the EQ-5D-5L.

All three HRQOL measures reflected a similar pattern: a decrease in quality of life with an increase in the number of comorbidities. This result is in accordance with previous studies [53,54]. However, only the VAS scores were statistically significant. This suggests that the VAS is more sensitive regarding the frequency of chronic health conditions.

As expected, we found a statistically significant positive correlation between the EQ-5D-5L index and VAS scores (0.61), which was consistent with previous studies [33,44,46,47] and indicates both measures’ reliability in estimating HRQoL. Patients “understood the meaning of the VAS scale and made reasonable choices based on their understanding”, as noted by [57]. In addition, we found a significant correlation between VAS and LSS (0.64) and a very strong significant correlation between EQ-5D-5L index and LSS (0.99), which was consistent with a previous study [33].

The agreement between the paired HRQoL measures was different. Our results showed excellent agreement between EQ-5D-5L index and LSS, poor to good agreement between the EQ-5D-5L index and VAS scores, and moderate to good agreement between the LSS and VAS values. The Bland–Altman plots confirmed these findings across the whole sample. However, some large differences emerged at the individual level. The results suggested that the EQ-5D-5L index and LSS measure may be used interchangeably. Previous investigations, as noted by [34], found substantial agreement and similar psychometric properties between the LSS and EQ-5D-5L index.

This is the first study of HRQoL in patients with dystonia using the EQ-5D-5L instrument and the Romanian value set. The foremost strength of our study lies in the applicability of the EQ-5D-5L and the utilization of the Romanian value set for calculating the EQ-5D-5L index and the analysis of three HRQoL measures (VAS, EQ-5D-5L index, and Level Sum Score) on the same data set.

The study of HRQoL in patients with dystonia is essential for greater understanding of the disorder’s effects, guiding patient-centered clinical management, and informing healthcare policy. Our study showed that problems with pain and discomfort were reported most frequently and had the most pronounced impact on HRQoL in patients with dystonia. The dispersion of patients’ profile data across numerous health profiles suggests that individualized treatment approaches may be necessary, thereby confirming findings from other studies [22,39,40].

Overall, the mean EQ-5D-5L index in this study was lower than the value for the broader Romanian population [32] and was lower those reported in other studies of neurological conditions. In addition, the VAS measure produced lower mean scores than the EQ-5D-5L index, which confirms the findings of a review of patients affected by neurological disease [51]. The EQ-5D-5L index and LSS measures showed the ability to detect differences between subgroups separated by factors such as age at the beginning of the survey, age of onset, type of disorder, and disease duration. Moreover, our study showed excellent agreement between the EQ-5D-5L index and LSS measure, in line with a previous study [34].

The EQ-5D-5L instrument has the potential utility for patients with neurological conditions, and the integration of HRQoL measurement using the EQ-5D-5L in dystonia-related clinical practice could be helpful for economic evaluation of dystonia interventions. Clinicians and policy makers can use the EQ-5D-5L instrument to track the progress of patients with dystonia throughout treatment and to compare the health status of different demographic groups. The EQ-5D-5L scale offers significant advantages in evaluating patients with dystonic movements due to its ease of administration by any competent physician or evaluator. This scale is crucial for monitoring patients’ health over time, providing a standardized method of assessing various aspects of health and determining the appropriate referral pathways.

The scale facilitates decision-making regarding patients’ referral either to a neurologist for botulinum toxin treatment, to a medical rehabilitation service, or to a psychologist. Specifically, if the score for pain/discomfort caused by dystonia is high, the patient should be referred for neurological revaluation and potential botulinum toxin treatment. If the mobility score is high, the patient should be directed to medical rehabilitation; if this is coupled with high pain scores, they should first see a neurologist before proceeding to rehabilitation. In cases with high self-care scores, referral to medical rehabilitation and home support is recommended. High scores for psychosocial problems, such as anxiety or depression, necessitate referral to a psychologist or psychiatrist.

The long-term application of the EQ-5D-5L scale enables dynamic evaluation of patients and assessment of treatment efficacy and its impact on quality of life. Furthermore, the profile of the studied group serves as a valuable reference for current evaluations, allowing clinicians to compare individual patient data with established benchmarks from the study. This comparative approach will enhance the precision and relevance of patient assessments, ultimately contributing to improved clinical outcomes.

Our study, however, has several limitations. Firstly, the sample size and the patient groups might have been insufficient, such that the apparent differences within small groups did not always reach statistical significance. For example, the imbalance between the number of male and female patients has an important influence on the HRQoL and can lead to highly variable results. Secondly, as our study is a cross-sectional study, any causal relationships remain to be demonstrated. Longitudinal research is needed to better explore this area. Thirdly, our study analyzed patients from only one medical center in one country (Romania). Thus, the findings cannot be generalized to all Romanian patients, even less so to patients from other countries.

Therefore, more research is needed to better understand the HRQoL in patients with dystonia. Further research could investigate the following important areas: (a) studies with larger sample sizes, with patients coming from several medical centers and ensuring the collection of several characteristics related to dystonia (e.g., clinical characteristics, etiology, treatment); (b) studies comparing EQ-5D-5L index values calculated with different national value sets in the same sample, in order to validate the EQ-5D-5L instrument and the methodology promoted by the EuroQOL Group; and (c) longitudinal studies using the EQ-5D-5L in order to identify changes in HRQoL values as a result of the treatments applied in the management and monitoring of patients with dystonia. Additionally, future studies should consider exploring the psychometric properties of the EQ-5D-5L and alternative HRQoL instruments (e.g., SF-36) in dystonia patients in order to demonstrate the validity and utility of these instruments.

## 5. Conclusions

Understanding the impact of dystonia on the quality of life is crucial in the development of a patient-centered approach to care. Beneficiaries include the patients themselves (through the acknowledgement of their specific needs) healthcare providers, researchers, and policy makers (through practical guidance on how they might allocate resources and design interventions).

To conclude, although we could find no directly comparable studies, our research findings support the applicability of the EQ-5D-5L instrument as a useful and reliable instrument for assessing the HRQoL of patients with dystonia. Future studies should consider exploring the psychometric properties of the EQ-5D-5L and other HRQoL instruments in dystonia patients. Our study is a contribution to the growing body of evidence concerning the impact of dystonia on the quality of life; it will serve to guide future research in this field.

## Figures and Tables

**Table 1:** Characteristics of the sample.

Characteristic	Total (N = 90)		Males (n = 22)		Females (n = 68)	
	N	%	N	%	N	%
	90	100.0	22	24.4	68	75.6
Age at the beginning of the survey (years), mean (SD)	58.72 (12.56)		55.59 (15.29)		59.74 (11.48)	
Age at onset (years), mean (SD)	48.76 (12.15)		44.23 (13.72)		50.21 (11.35)	
Disease duration (years), mean (SD)	9.86 (7.74)		11.18 (10.74)		9.43 (6.53)	
Comorbidities						
None	23	25.6	7	31.8	16	23.5
One	34	37.8	8	36.4	26	38.2
Two or more	33	36.7	7	31.8	26	38.2
Type of disorder						
Dystonia	72	80.0	18	81.8	54	79.4
Hemispasm	18	20.0	4	18.2	14	20.6
Body distribution						
Focal	78	86.7	19	86.4	59	86.8
Segmental	10	11.1	3	13.6	7	10.3
Multifocal	1	1.1	0	0.0	1	1.5
Generalized	1	1.1	0	0.0	1	1.5
Type of botulinum toxin						
Xeomin	34	37.8	10	45.5	24	35.3
Dysport	56	62.2	12	55.5	44	64.7

**Table 2:** Most frequently reported health states.

Health State	Total (n = 90)		Male (n = 22)		Female (n = 68)	
	Frequency	%	Frequency	%	Frequency	%
11111	9	10.0	4	18.2	5	7.4
11121	5	5.6	1	4.5	4	5.9
11122	3	3.3	1	4.5	2	2.9
21233	3	3.3	1	4.5	2	2.9
33333	3	3.3	1	4.5	2	2.9
11112	2	2.2	0	0.0	2	2.9
11133	2	2.2	0	0.0	2	2.9
11222	2	2.2	0	0.0	2	2.9
11231	2	2.2	0	0.0	2	2.9
21232	2	2.2	0	0.0	2	2.9
22231	2	2.2	1	4.5	1	1.5
44555	2	2.2	1	4.5	1	1.5

**Table 3:** EQ-5D-5L frequencies and proportions reported via the metrics of dimension and level.

	Mobility	Selfcare	Activity	Pain	Anxiety
	n (%)	n (%)	n (%)	n (%)	n (%)
Level 1 (No problems)	37 (41.1)	59 (65.6)	30 (33.3)	16 (17.8)	32 (35.6)
Level 2 (Slight problems)	22 (24.5)	11 (12.1)	27 (30.0)	21 (23.2)	26 (28.7)
Level 3 (Moderate problems)	13 (14.4)	10 (11.1)	20 (22.2)	32 (35.6)	22 (24.5)
Level 4 (Severe problems)	13 (14.4)	5 (5.6)	8 (8.9)	14 (15.6)	5 (5.6)
Level 5 (Extreme problems/incapacity)	5 (5.6)	5 (5.6)	5 (5.6)	7 (7.8)	5 (5.6)

**Table 4:** Reported problems in dimensions stratified by age groups for all patients.

EQ-5D-5L	Level	Age Group, Years						All Patients	
Dimension		≤54		55–64		65+			
		n	%	n	%	n	%	n	%
Total		31	34.4	22	24.5	37	41.1	90	100
Mobility									
	No problems	11	35.5	9	40.9	17	45.9	37	41.1
	Slight problems	7	22.6	4	18.2	11	29.7	22	24.5
	Moderate problems	6	19.4	4	18.2	3	8.1	13	14.4
	Severe problems	2	6.5	5	22.7	6	16.2	13	14.4
	Unable to	5	16.1	0	0.0	0	0.0	5	5.6
	Any problems (levels 2–5)	20	64.5	13	59.1	20	54.1	53	58.9
Self-care									
	No problems	16	51.6	15	68.2	28	75.7	59	65.6
	Slight problems	5	16.1	2	9.1	4	10.8	11	12.1
	Moderate problems	5	16.1	3	13.6	2	5.4	10	11.1
	Severe problems	1	3.2	1	4.5	3	8.1	5	5.6
	Unable to	4	12.9	1	4.5	0	0.0	5	5.6
	Any problems (levels 2–5)	15	48.4	7	31.8	9	24.3	31	34.4
Usual activities									
	No problems	3	9.7	8	36.4	19	51.4	30	33.3
	Slight problems	12	38.7	5	22.7	10	27.0	27	30.0
	Moderate problems	10	32.3	6	27.3	4	10.8	20	22.2
	Severe problems	2	6.5	3	13.6	3	8.1	8	8.9
	Unable to	4	12.9	0	0.0	1	2.7	5	5.6
	Any problems (levels 2–5)	28	90.3	14	63.6	18	48.6	60	66.7
Pain/discomfort									
	None	3	9.7	5	22.7	8	21.6	16	17.8
	Slight	6	19.4	4	18.2	11	29.7	21	23.2
	Moderate	14	45.2	6	27.3	12	32.4	32	35.6
	Severe	6	19.4	4	18.2	4	10.8	14	15.6
	Extreme	2	6.5	3	13.6	2	5.4	7	7.8
	Any problems (levels 2–5)	28	90.3	17	77.3	29	78.4	74	82.2
Anxiety/depression									
	None	8	25.8	7	31.8	17	45.9	32	35.6
	Slight	11	35.5	5	22.7	10	27.0	26	28.7
	Moderate	7	22.6	8	36.4	7	18.9	22	24.5
	Severe	3	9.7	1	4.5	1	2.7	5	5.6
	Extreme	2	6.5	1	4.5	2	5.4	5	5.6
	Any problems (levels 2–5)	23	74.2	15	68.2	20	54.1	58	64.4

**Table 5:** Reported problems in dimensions stratified by age groups for male and female patients.

EQ-5D-5L	Male								Female							
Dimension	Age Groups, Years						Total		Age Groups, Years						Total	
	≤54		55–64		65+				≤54		55–64		65+			
	n	%	n	%	n	%	n	%	n	%	n	%	n	%	n	%
Total	10	45.4	4	18.2	8	36.4	22	24.4	21	30.9	18	26.5	29	42.6	68	75.6
Mobility																
None	4	40.0	2	50.0	3	37.5	9	40.9	7	33.3	7	38.9	14	48.3	28	41.2
Slight	0	0.0	1	25.0	3	37.5	4	18.2	7	33.3	3	16.7	8	27.6	18	26.5
Moderate	2	20.0	1	25.0	0	0.0	3	13.6	4	19.0	3	16.7	3	10.3	10	14.7
Severe	2	20.0	0	0.0	2	25.0	4	18.2	0	0.0	5	27.8	4	13.8	9	13.2
Unable to	2	20.0	0	0.0	0	0.0	2	9.1	3	14.3	0	0.0	0	0.0	3	4.4
Any problems	6	60.0	2	50.0	5	62.5	13	59.1	14	66.7	11	61.1	15	51.7	40	58.8
Self-Care																
None	5	50.0	4	100.0	6	75.0	15	68.2	11	52.4	11	61.1	22	75.9	44	64.7
Slight	1	10.0	0	0.0	2	25.0	3	13.6	4	19.0	2	11.1	2	6.9	8	11.8
Moderate	1	10.0	0	0.0	0	0.0	1	4.5	4	19.0	3	16.7	2	6.9	9	13.2
Severe	1	10.0	0	0.0	0	0.0	1	4.5	0	0.0	1	5.6	3	10.3	4	5.9
Unable to	2	20.0	0	0.0	0	0.0	2	9.1	2	9.5	1	5.6	0	0.0	3	4.4
Any problems	5	50.0	0	0.0	2	25.0	7	31.8	10	47.6	7	38.9	7	24.1	24	35.3
Activities																
None	2	20.0	3	75.0	3	37.5	8	36.4	1	4.8	5	27.8	16	55.2	22	32.4
Slight	2	20.0	1	25.0	3	37.5	6	27.2	10	47.6	4	22.2	7	24.1	21	30.9
Moderate	3	30.0	0	0.0	1	12.5	4	18.2	7	33.3	6	33.3	3	10.3	16	23.5
Severe	1	10.0	0	0.0	1	12.5	2	9.1	1	4.8	3	16.7	2	6.9	6	8.8
Unable to	2	20.0	0	0.0	0	0.0	2	9.1	2	9.5	0	0.0	1	3.4	3	4.4
Any problems	8	80.0	1	25.0	5	62.5	14	63.6	20	95.2	13	82.2	13	44.8	46	67.6
Pain																
None	2	20.0	1	25.0	1	12.5	4	18.2	1	4.8	4	22.2	7	24.1	12	17.6
Slight	0	0.0	2	50.0	3	37.5	5	22.7	6	38.1	2	11.1	8	27.6	18	26.5
Moderate	3	30.0	1	25.0	1	12.5	5	22.7	11	52.4	5	27.8	11	37.9	27	39.7
Severe	3	30.0	0	0.0	3	37.5	6	27.3	3	14.3	4	22.2	1	3.4	8	11.8
Extreme	2	20.0	0	0.0	0	0.0	2	9.1	0	0.0	3	16.7	2	6.9	5	7.4
Any problems	8	80.0	3	75.0	7	87.5	18	81.8	20	95.2	14	77.8	22	75.9	58	82.6
Anxiety																
None	2	20.0	2	50.0	4	50.0	8	36.4	6	28.6	5	27.8	13	44.8	24	35.3
Slight	4	40.0	1	25.0	3	37.5	8	36.4	7	33.3	4	22.2	7	24.1	18	26.5
Moderate	2	20.0	1	25.0	0	0.0	3	13.6	5	23.8	7	38.9	7	24.1	19	27.9
Severe	0	0.0	0	0.0	1	12.5	1	4.5	3	14.3	1	5.6	0	0.0	4	5.9
Extreme	2	20.0	0	0.0	0	0.0	2	9.1	0	0.0	1	5.6	2	6.9	3	4.4
Any problems	8	80.0	2	50.0	4	50.0	14	63.6	15	71.4	13	72.2	16	55.2	44	64.7

**Table 6:** Shannon index (*H*′) and Shannon evenness index (*J*′) for EQ-5D-5L.

	EQ-5D-5L	Dimension				
		Mobility	Self-Care	Usual Activity	Pain/Discomfort	Anxiety/Depression
*H*′	5.73	2.06	1.59	2.07	2.17	2.01
*J*′	0.49	0.89	0.68	0.89	0.93	0.86

**Table 7:** Descriptive statistics of the HRQoL measures according to demographic characteristics.

		VAS		EQ-5D-5L Index		LSS	
	n (%)	Mean (SD)	Median (IQR)	Mean (SD)	Median (IQR)	Mean (SD)	Median (IQR)
Overall	90 (100)	0.61 (0.21)	0.60 (0.49–0.80)	0.74 (0.26)	0.81 (0.68–0.91)	0.70 (0.24)	0.75 (0.50–0.90)
Sex							
Male	22 (24.4)	0.59 (0.25)	0.60 (0.40–0.80)	0.70 (0.34)	0.80 (0.62–0.92)	0.68 (0.29)	0.77 (0.49–0.91)
Female	68 (75.6)	0.62 (0.20)	0.60 (0.50–0.79)	0.75 (0.23)	0.81 (0.68–0.91)	0.70 (0.23)	0.75 (0.51–0.90)
*p*-value ^a^		0.745		0.866		0.974	
Age at start survey, years							
≤54	31 (34.5)	0.60 (0.22)	0.60 (0.45–0.75)	0.66 (0.30)	0.77 (0.58–0.84)	0.62 (0.25)	0.65 (0.50–0.80)
55–64	22 (24.4)	0.62 (0.23)	0.65 (0.44–0.72)	0.74 (0.24)	0.80 (0.58–0.91)	0.69 (0.24)	0.72 (0.49–0.87)
65+	37 (41.1)	0.61 (0.20)	0.60 (0.50–0.80)	0.80 (0.21)	0.86 (0.78–0.93)	0.77 (0.22)	0.85 (0.70–0.90)
*p*-value ^b^		0.980		0.025		0.023	
Age at onset, years							
≤40	22 (24.5)	0.65 (0.25)	0.60 (0.50–0.90)	0.71 (0.31)	0.79 (0.66–0.88)	0.68 (0.26)	0.72 (0.50–0.86)
41–55	38 (42.2)	0.56 (0.19)	0.52 (0.40–0.70)	0.69 (0.25)	0.78 (0.56–0.87)	0.64 (0.24)	0.70 (0.45–0.85)
55+	30 (33.3)	0.65 (0.21)	0.67 (0.50–0.81)	0.82 (0.21)	0.88 (0.78–0.95)	0.78 (0.22)	0.85 (0.69–0.95)
*p*-value ^b^		0.110		0.020		0.023	
Comorbidities							
None	23 (25.5)	0.67 (0.21)	0.70 (0.60–0.80)	0.76 (0.29)	0.83 (0.70–0.94)	0.73 (0.25)	0.90 (0.65–0.90)
One	34 (37.8)	0.64 (0.21)	0.60 (0.50–0.81)	0.75 (0.26)	0.83 (0.70–0.92)	0.72 (0.24)	0.77 (0.61–0.91)
Two or more	33 (36.7)	0.54 (0.20)	0.50 (0.35–0.70)	0.71 (0.24)	0.78 (0.58–0.88)	0.65 (0.24)	0.75 (0.65–0.85)
*p*-value ^b^		0.029		0.253		0.357	
Type of disorder							
Dystonia	72 (80.0)	0.59 (0.21)	0.60 (0.45–0.70)	0.71 (0.26)	0.79 (0.65–0.88)	0.66 (0.24)	0.70 (0.50–0.85)
Hemispasm	18 (20.0)	0.70 (0.20)	0.70 (0.54–0.91)	0.84 (0.21)	0.91 (0.81–0.97)	0.83 (0.20)	0.90 (0.75–0.96)
*p*-value ^a^		0.049		0.004		0.003	
Disease duration, years							
≤8	49 (54.4)	0.60 (0.20)	0.60 (0.47–0.70)	0.69 (0.30)	0.80 (0.62–0.89)	0.66 (0.26)	0.75 (0.50–0.85)
9 to 14	26 (28.9)	0.68 (0.20)	0.70 (0.50–0.80)	0.85 (0.13)	0.87 (0.78–0.95)	0.80 (0.17)	0.82 (0.74–0.95)
15 or more	15 (16.7)	0.53 (0.23)	0.50 (0.30–0.60)	0.69 (0.23)	0.78 (0.55–0.81)	0.63 (0.24)	0.70 (0.35–0.80)
*p*-value ^b^		0.062		0.024		0.029	

^a^ Mann–Whitney U test; ^b^ Kruskal–Wallis H test.

**Table 8:** Correlations and agreement between the HRQoL measures.

Measures of Association and Agreement	EQ-5D-5L and LSS	EQ-5D-5L and VAS	LSS and VAS
Spearman rank correlation, *rho*	0.991 **	0.616 **	0.636 **
(95% CI for *rho*)	(0.987, 0.994)	(0.468, 0.630)	(0.493, 0.745)
ICC	0.970	0.683	0.789
95% of ICC	(0.934, 0.984)	(0.338, 0.820)	(0.559, 0.862)
Mean differences (SE)	0.040 (0.008)	0.127 (0.022)	0.087 (0.020)
95% CI for mean difference	(0.024, 0.056)	(0.082, 0.172)	(0.048, 0.126)
Lower limits of agreement	−0.109	−0.292	−0.278
95% CI	(−0.137, −0.081)	(−0.369, −0.215)	(−0.345, −0.211)
Upper limits of agreement	0.190	0.545	0.451
95% CI	(0.162, 0.218)	(0.468, 0.622)	(0.384, 0.518)

** Correlation is significant at the 0.01 level (two-tailed).

## Data Availability

The datasets presented in this article are not readily available because the authors do not own the database used in the presented article. They requested access to the database from the owner, the institution that conducted the study. The owner decided to keep the database private. Requests to access the datasets should be directed to the owner of the dataset.

## Abbreviations

HRQoL	Health-related quality of life
QoL	Quality of life
EQ-5D	EuroQoL five-dimension questionnaire
EQ-5D-5L	EuroQoL five-dimension questionnaire five-level
EQ-5D-3L	EuroQoL five-dimension questionnaire three-level
VAS	Visual analog scale
LSS	Level sum score
MDS	Movement Disorder Society
PD	Parkinson’s disease
MS	Multiple sclerosis
CD	Cervical dystonia
SF-36	Short-form 36-item health profile
IQR	Interquartile range
ICC	Intraclass correlation coefficient
SD	Standard deviation
